# Intelligence

**Published:** 1809-01

**Authors:** 


### INTELLIGENCE.

Mr. J. Pick, of Ipswich, has lately analyzed a stone of the calcareous species, frequently met with in that part of the country, and called by the common people thunder-pick, from the supposition of its falling from the clouds in storms of thunder and lightning. It occurs in crystals weighing from 40 to 100 grains, of a conic shape, with a cavity at the base, extending about a fourth part down to the centre of the crystal. Its colour arises from grey, brown, brownish red, to almost black, semi-transparent. They are generally discovered solitary by the husbandmen when at plough, or turning up the earth in any other way. When scratched with a knife, this stone has a strong alliaceous or urinous smell. Its cross fracture is fibrous, with the striæ diverging nearly as from a common centre. Its longitudinal fracture is glittering, with the striæ parallel. It is moderately hard, and of the specific gravity of 2,663. Its properties, as ascertained by the examinate, are as follow:—1. When heated upon charcoal before the blow-pipe, its colour disappears, but it is infusible. 2. With phosphate of soda, it is difficultly soluble, and fuses into an enamelled bead. 3. With borate of soda it dissolves more readily, and fuses into a semi-translucid white globule. 4. With caustic soda it could be only partially fused into a white enamel. 5. The substances of which, by analysis, 100 grains were found to be composed, are:

| | | |
|---|---|---|
| Carbonic acid gas - - - - - - - | 43,55 | grs. |
| Lime - - - - - - - - - - - - - - | 53,95 | |
| Oxides of manganese and iron - - | 40 | |
| Water and loss - - - - - - - - - | 2,10 | |
| | 100 | |

The Honourable Basil Corcoran, of Portman Square, has made considerable improvements in warm water, vapour, and air baths. They are constructed in his own house, and the many complaints he has been enabled to remove by them, have induced him to prepare a model and engraving, for the further benefit of the public.

The

The Chinese, instead of raising their fruit-trees from seeds or grafts, as is the practice in Europe, adopt the following method:—They select a branch fit for the purpose, and round it they wind a rope of straw besmeared with cow-dung, until a ball is formed five or six times the diameter of the branch. Immediately under this ball, they divide the bark down to the wood, for nearly two-thirds of the circumference of the branch. A cocoa-nut shell, or small pot, is hung over the ball, with a hole in its bottom, so small that water put into it will only fall in drops. By this the rope is kept constantly moist, a circumstance necessary to the easy admission of young roots. In about three weeks it is supposed that some of the roots have struck into the rope, when the remainder of the bark is cut, and the former incision carried deeper into the wood; it is repeated in three weeks more. In about two months, the roots are seen intersecting each other on the surface of the ball, which is a sign that they are sufficiently advanced to admit of the separation of the branch from the tree, which is done by sawing at the incision, taking care not to cut off the rope, which by this time is rotten, and the branch is planted as a young tree. It is probable that a month longer would be necessary for the operation in England, from the difference of climate; but by this means, when the branches are large, three or four years are sufficient to bring them to a state of full bearing. Timber-trees, it is supposed, may be advantageously propagated in the same way.

---

Dr. HERDMAN has just published A Letter, addressed to the Lord Bishop of Durham and the other Members of the General Committee of the Society for bettering the Condition and increasing the Comforts of the Poor,' proposing a Plan for improving Dispensaries, and the Medical Treatment of the diseased Poor.

About the middle of this month will be published, in two vols. 8vo. Outlines of Mineralogy, containing a general history of the principal varieties of mineral substances; together with a particular statement of their physical characters, and chemical analysis; by J. KIDD, M. D. Professor of Chemistry in the University of Oxford.

The Medical and Chirurgical Society of London will shortly publish the first volume of their Records. It will contain some very valuable contributions from practitioners of first-rate eminence in the metropolis.

Mr. ARNOLD, of Leicester, has just put to press a valuable practical volume of Observations on the Management of the Insane; a subject on which thirty years experience has eminently qualified him to write.

---

We have to lament, in common with the whole scientific world, the death of one of our highly valued Correspondents, Dr. Beddoes; who died on the 24th of December, at his house at Clifton, after a lingering illness.

### *Diseases and Casualties in London in the Year* 1808.

| | |
|---|---|
| Abortive and Stillborn | 462 |
| Abscess | 49 |
| Aged | 1555 |
| Ague | 5 |
| Apoplexy & Suddenly | 229 |
| Asthma and Phthisic | 586 |
| Bedridden | 3 |
| Bleeding | 28 |
| Bursten and Rupture | 26 |
| Cancer | 54 |
| Canker | 2 |
| Chicken Pox | 3 |
| Childbed | 172 |
| Colds | 11 |
| Colic, Gripes, &c. | 19 |
| Consumption | 5220 |
| Convulsions | 4164 |
| Cough, and Hooping Cough | 326 |
| Cow Pox | 1 |
| Croup | 76 |
| Diabetes | 2 |
| Dropsy | 870 |
| Evil | 8 |
| Fevers of all kinds | 1168 |
| Fistula | 1 |
| Flux | 10 |
| French Pox | 28 |
| Gout | 33 |
| Gravel, Stone, and Strangury | 18 |
| Grief | 5 |
| Headmouldshot, Horseshoehead, and Water in the Head | 193 |
| Jaundice | 39 |
| Jaw Locked | 2 |
| Inflammation | 765 |
| Lethargy | 1 |
| Livergrown | 14 |
| Lunatick | 172 |
| Measles | 1386 |
| Miscarriage | 2 |
| Mortification | 200 |
| Palsy | 98 |
| Piles | 1 |
| Pleurisy | 17 |
| Purples | 1 |
| Quinsy | 3 |
| Rheumatism | 7 |
| Scurvy | 2 |
| Small Pox | 1169 |
| Sore Throat | 9 |
| Sores and Ulcers | 5 |
| Spasm | 15 |
| St. Vitus's Dance | 1 |
| Stoppage in the Stom. | 12 |
| Teeth | 319 |
| Thrush | 48 |
| Tumour | 1 |
| Worms | 3 |

| | |
|---|---|
| Bit by a Mad Cat | 1 |
| Bit by Mad Dogs | 3 |
| Bruised | 1 |
| Burnt | 51 |
| Drowned | 123 |
| Excessive Drinking | 7 |
| Found Dead | 17 |
| Fractured | 2 |
| Frighted | 1 |
| Frozen | 2 |
| Killed by Falls and several other Accidents | 77 |
| Killed themselves | 36 |
| Poisoned | 3 |
| Scalded | 5 |
| Starved | 2 |
| Suffocated | 4 |
| Total | 335 |

| | | | |
|---|---|---|---|
| Christened | Males | 10189 | In all 19906 |
| | Females | 9717 | |
| Buried | Males | 10228 | In all 19954 |
| | Females | 9726 | |

Whereof have died,

| | |
|---|---|
| Under Two Years of Age | 6075 |
| Between Two and Five | 2466 |
| Five and Ten | 847 |
| Ten and Twenty | 643 |
| Twenty and Thirty | 1200 |
| Thirty and Forty | 1792 |
| Forty and Fifty | 1971 |
| Fifty and Sixty | 1690 |
| Sixty and Seventy | 1499 |
| Seventy and Eighty | 1200 |
| Eighty and Ninety | 504 |
| Ninety and a Hundred | 65 |
| A Hundred | 1 |
| A Hundred and Two | 1 |

Increased in the Burials this Year 1620.

This Register exhibits a considerable increase in the proportion of deaths under two years of age, the whole number of deaths exceeding the preceding year only 1620, and the mortality of children under two years of age greater by nearly [illegible]00 than in either 1806, or 1807. This may be accounted for by the great increase of measles, which exceeds every thing that can be produced in any former years. It is also well known that inflammatory diseases have been more severe and frequent than usual. The Yearly Bills, inaccurate as they are, confirm this; as well as most other facts on a large scale. The number of deaths by Croup is greater than in either of the two preceding years; and under the general term of "Inflammation," the difference is not less remarkable. The deaths by Small-pox are 128 less than last year, and 11 more than the preceding year.

---

Dr. Buxton's Lectures on the Theory and Practice of Medicine, and on Materia Medica, will be commenced about the 20th of January, 1809, at the London Hospital.—For particulars apply to to Mr. Price, Apothecary at the Hospital, or to Dr. Buxton, Fenchurch Street.

Mr. BROOKES will commence his Spring Course of Lectures on Anatomy, Physiology, and Surgery, on Saturday the 21st of January, at two o'clock, at the Theatre of Anatomy, Blenheim Street, Great Malborough Street.—The Dissecting Rooms are open all the morning, where Mr. Brooks attends.

Dr. CLARKE and Mr. CLARKE will begin their Spring Course of Lectures on Midwifery, and the Diseases of Women and Children, on Thursday, January 26. The Lectures are read every day, at the house of Mr. Clarke, No. 10, Upper John-street, Golden Square, from a quarter past ten o'clock in the morning, till a quarter past eleven, for the convenience of students attending the Hospitals.

Dr. CLOUGH, 68, Berners Street, will on Monday the 9th of January, 1809, at ten o'clock in the morning, resume his Course of Lectures on Puerperal Anatomy, Physiology, and Pathology.

Dr. REID's Lectures on the Theory and Practice of Medicine will commence on Monday the 23d of January, at nine o'clock in the morning, at his House, Grenville Street, Brunswick Square.

Dr. SQUIRE will, on the 4th of January, 1809, begin a Course of Lectures on the Theory and Practice of Midwifery, and the Diseases of Women and Children.

Mr. TAUNTON's Lectures on Anatomy, Physiology, Pathology, and Surgery, will commence on Saturday January the 21st, 1809, at eight o'clock in the Evening *precisely*, and be continued every Tuesday, Thursday, and Saturday, at the same hour.—Particulars may be had by applying to Mr. Taunton, Greville Street, Hatton-Garden.

